# 

**Published:** 1883-10

**Authors:** 


WHOLE NUMBER, CCLVII.

OCTOBER, 1883.

NUMBER 3, VOLUME XXIII.

### THE BUFFALO Medical and Surgical Journal.

INDEXED

EDITORS:

THOS. LOTHROP, M. D., A. R. DAVIDSON, M. D., P. W. VAN PEYMA, M. D.

PUBLISHED BY THE MEDICAL JOURNAL ASSOCIATION.

No. 5 WEST CHIPPEWA ST.

Entered at the Post-Office at Buffalo, N. Y., as Second-class Mail Matter.

